# Supplementary material for: Does patients’ age predict their clinical outcomes following non-infectious epiglottitis? A systematic review
Source: PLoS One. 2025 Feb 10;20(2):e0318648. doi: 10.1371/journal.pone.0318648 (PMC11809843; doi:10.1371/journal.pone.0318648)
Supplement: S1 Table — (DOCX) [file pone.0318648.s001.docx]

**S1 Table. The detailed search criteria employed in the literature search**

| Database | No. | Search Query | Results |
| --- | --- | --- | --- |
| PubMed [date of search: 22/12/2023] | | | |
|  | #1 | Epiglottitis[tiab] | 1475 |
|  | #2 | Noninfectious OR non-infectious OR trauma OR traumatic OR “foreign body” OR “foreign object” OR inhal* OR foreign OR chemical OR burn OR thermal OR heat OR smok* OR cocaine OR wound OR injur* OR stab OR gunshot | 6077291 |
|  | #3 | "Pediatrics"[Mesh] OR "Child"[Mesh] OR "Adolescent"[Mesh] OR "Infant"[Mesh] OR child* OR pediatric* OR paediatric* OR adolescent* OR infant* OR month* OR age* OR years OR adult* OR "Adult"[Mesh] OR "Age Groups"[Mesh] OR old | 14000311 |
|  | #4 | #1 AND #2 AND #3 | 198 |
| Scopus [date of search: 22/12/2023] | | | |
|  | #1 | TITLE-ABS-KEY (Epiglottitis) | 2937 |
|  | #2 | ALL (Noninfectious) OR ALL (non-infectious) OR ALL (trauma) OR ALL (traumatic) OR ALL (“foreign body”) OR ALL (“foreign object”) OR ALL (inhal*) OR ALL (foreign) OR ALL (chemical) OR ALL (burn) OR ALL (thermal) OR ALL (heat) OR ALL (smok*) OR ALL (cocaine) OR ALL (wound) OR ALL (injur*) OR ALL (stab) OR ALL (gunshot) | 29318790 |
|  | #3 | ALL (child*) OR ALL (pediatric*) OR ALL (paediatric*) OR ALL (adolescent*) OR ALL (infant*) OR ALL (month*) OR ALL (age*) OR ALL (years) OR ALL (adult*) OR ALL (old) | 40294413 |
|  | #4 | #1 AND #2 AND #3 | 661 |
| Web of Science [date of search: 22/12/2023] | | | |
|  | #1 | AB=Epiglottitis | 502 |
|  | #2 | ALL=Noninfectious OR ALL=non-infectious OR ALL=trauma OR ALL=traumatic OR ALL=“foreign body” OR ALL=“foreign object” OR ALL=inhal* OR ALL=foreign OR ALL=chemical OR ALL=burn OR ALL=thermal OR ALL=heat OR ALL=smok* OR ALL=cocaine OR ALL=wound OR ALL=injur* OR ALL=stab OR ALL=gunshot | 10776847 |
|  | #3 | ALL=child* OR ALL=pediatric* OR ALL=paediatric* OR ALL=adolescent* OR ALL=infant* OR ALL=month* OR ALL=age* OR ALL=years OR ALL=adult* OR ALL=old | 16689293 |
|  | #4 | #1 AND #2 AND #3 | 81 |
| Google Scholar [date of search: 22/12/2023] | | | |
|  | With all of the words | Epiglottitis age/yeasr/old /months | - |
|  | With the exact phrase | - | - |
|  | With at least one of the words | Noninfectious non-infectious trauma traumatic foreign object inhal* chemical burn thermal heat smok* cocaine wound injur* stab gunshot | - |
|  | Total |  | 200 |
