# Supplementary material for: Does patients’ age predict their clinical outcomes following non-infectious epiglottitis? A systematic review
Source: PLoS One. 2025 Feb 10;20(2):e0318648. doi: 10.1371/journal.pone.0318648 (PMC11809843; doi:10.1371/journal.pone.0318648)
Supplement: S2 Table — (DOCX) [file pone.0318648.s002.docx]

**S2 Table. The methodological quality of included studies based on the NIH quality assessment tool for case series/case reports.**

| Study | Was the study question or objective clearly stated? | Was the study population clearly and fully described, including a case definition? | Were the cases consecutive? | Were the subjects comparable? | Was the intervention clearly described? | Were the outcome measures clearly defined, valid, reliable, and implemented consistently across all study participants? | Was the length of follow-up adequate? | Were the statistical methods well described? | Were the results well described? | Quality rating |
| --- | --- | --- | --- | --- | --- | --- | --- | --- | --- | --- |
| Alkaabi (2023) | Yes | Yes | N/A | N/A | Yes | Yes | Yes | NR | Yes | Good |
| Alpay (2008) | Yes | Yes | N/A | N/A | Yes | Yes | Yes | NR | Yes | Good |
| Alpay (2009) | Yes | Yes | N/A | N/A | Yes | Yes | Yes | NR | Yes | Good |
| Ames (2000) | Yes | Yes | N/A | N/A | Yes | Yes | Yes | NR | Yes | Fair |
| Asrar (2005) | Yes | Yes | N/A | N/A | Yes | Yes | Yes | NR | Yes | Fair |
| Boyden (1970) | Yes | Yes | Yes | Yes | Yes | Yes | Yes | Yes | Yes | Good |
| Bozzella (2020) | Yes | Yes | N/A | N/A | Yes | Yes | Yes | NR | Yes | Good |
| Carmel-Neiderman (2018) | Yes | Yes | N/A | N/A | Yes | Yes | Yes | NR | Yes | Good |
| Chung (2000) | Yes | Yes | N/A | N/A | Yes | Yes | Yes | NR | Yes | Good |
| Chung (2002) | Yes | Yes | N/A | N/A | Yes | Yes | Yes | NR | Yes | Good |
| Dalrymple (2020) | Yes | Yes | N/A | N/A | Yes | Yes | Yes | NR | Yes | Good |
| Doyle (2023) | Yes | Yes | N/A | N/A | Yes | Yes | Yes | NR | Yes | Good |
| Garg (2020) | Yes | Yes | N/A | N/A | Yes | Yes | Yes | NR | Yes | Good |
| Harjacek (1992) | Yes | Yes | N/A | N/A | Yes | Yes | Yes | NR | Yes | Good |
| Inaguma (2019) | Yes | Yes | N/A | N/A | Yes | Yes | Yes | NR | Yes | Good |
| Kabbani (1995) | Yes | Yes | N/A | N/A | Yes | Yes | Yes | NR | Yes | Good |
| Kavanagh (2008) | Yes | Yes | N/A | N/A | Yes | Yes | Yes | NR | Yes | Good |
| Khorrami (2023) | Yes | Yes | N/A | N/A | Yes | Yes | Yes | NR | Yes | Good |
| Kornak (1996) | Yes | Yes | Yes | Yes | Yes | Yes | Yes | Yes | Yes | Good |
| Kudchadkar (2014) | Yes | Yes | N/A | N/A | Yes | Yes | Yes | NR | Yes | Good |
| Lai (2000) | Yes | Yes | Yes | Yes | Yes | Yes | Yes | Yes | Yes | Good |
| Lichtor (2016) | Yes | Yes | N/A | N/A | Yes | Yes | Yes | NR | Yes | Good |
| Lu (2023) | Yes | Yes | N/A | N/A | Yes | Yes | Yes | NR | Yes | Good |
| Mayo-Smith (1997) | Yes | Yes | Yes | Yes | Yes | Yes | Yes | Yes | Yes | Good |
| McKinney (1995) | Yes | Yes | N/A | N/A | Yes | Yes | Yes | NR | Yes | Good |
| O'Bier (2005) | Yes | Yes | N/A | N/A | Yes | Yes | Yes | NR | Yes | Fair |
| Parsons (1996) | Yes | Yes | Yes | Yes | Yes | Yes | Yes | Yes | Yes | Good |
| Rao (2021) | Yes | Yes | N/A | N/A | Yes | Yes | Yes | NR | Yes | Good |
| Savitt (1991) | Yes | Yes | N/A | N/A | Yes | Yes | Yes | NR | Yes | Good |
| Tsai (2014) | Yes | Yes | N/A | N/A | Yes | Yes | Yes | NR | Yes | Good |
| Verhees (2018) | Yes | Yes | N/A | N/A | Yes | Yes | Yes | NR | Yes | Good |
| Vinod (2007) | Yes | Yes | N/A | N/A | Yes | Yes | Yes | NR | Yes | Good |
| Yokoyama (2018) | Yes | Yes | N/A | N/A | Yes | Yes | Yes | NR | Yes | Good |
| Orhan (2015) | Yes | Yes | Yes | Yes | Yes | Yes | Yes | Yes | Yes | Good |
| Bonadio (1991) | Yes | Yes | Yes | Yes | Yes | Yes | Yes | Yes | Yes | Good |
| Deutsch (2004) | Yes | Yes | N/A | N/A | Yes | Yes | Yes | NR | Yes | Good |
| Yen (2003) | Yes | Yes | N/A | N/A | Yes | Yes | Yes | NR | Yes | Fair |
| Shenoy (2009) | Yes | Yes | N/A | N/A | Yes | Yes | Yes | NR | Yes | Fair |
| Laufkotter (1989) | Yes | Yes | N/A | N/A | Yes | Yes | Yes | NR | Yes | Good |
| Watts (1996) | Yes | Yes | N/A | N/A | Yes | Yes | Yes | NR | Yes | Fair |
