# Supplementary material for: Does patients’ age predict their clinical outcomes following non-infectious epiglottitis? A systematic review
Source: PLoS One. 2025 Feb 10;20(2):e0318648. doi: 10.1371/journal.pone.0318648 (PMC11809843; doi:10.1371/journal.pone.0318648)
Supplement: Attachment — Submitted filename: RESPONSE TABLE.docx [file pone.0318648.s007.docx]

**RESPONSE TABLE**

Manuscript ID: PONE-D-24-03809

Manuscript Title: Does Patients’ Age Predict Their Clinical Outcomes Following Non-Infectious Epiglottitis? A Systematic Review

| **Raised Comment** | **Authors’ Response** | **Applied Changes** |
| --- | --- | --- |
| Section 2.3- What was the decision where diagnosis was in doubt? | Thank you for your question.  At first, patients were closely monitored in the ED or ICU (if clinical condition was rapidly deteriorating).  Empirical antibiotics were given along with corticosteroids to manage the inflammation and prevent 2ry infection (blood cultures were drawn before antibiotics were given).  Additional diagnostic procedures were used to confirm the diagnosis, including repeated laryngoscopy, CT, or MRI.  If the study did not clearly report the diagnostic approach, it was deemed ineligible. | Under section 2.3, we added the following sentence: “When diagnosis was in doubt, patients were closely monitored in the Emergency department or ICU, administered empirical antibiotic therapy plus corticosteroid (to prevent secondary infection), and underwent further diagnostic procedures such as repeated laryngoscopy, CT, or MRI. Records were excluded if they reported infectious epiglottitis or lacked data on patients’ age, etiology of epiglottitis, or clinical outcomes. Additionally, if a study did not clarify the diagnostic approach to confirm the diagnosis, it was ruled out.” |
| 3.5- please give range of ICU stay length | Thank you for your comment. We added it to the manuscript. The ICU stay in the whole population ranged from 1 to 13 days. | Under section 3.5, we added this: “(SD = 3.25, range 1-13)” |
| 4- 160% is a difference rather than an increase, as is 350%; please modify. | Thank you for your comment. It was calculated as the percent difference/change.  This has been updated in the manuscript.  In the Abstract, as to avoid confusing the readers, we just provided the raw % for each group (children vs adults) instead of using the % difference. | We replaced “% increase” with “% difference”.  In the abstract, we updated the results section as follows: “Twenty-three children and 34 adults were analyzed. The presentation with stridor (56.52% vs. 14.7%), drooling (56.52% vs. 26.47%), cyanosis (17.39% vs. 0%), and sternal retraction (13.04% vs. 0%) was more common among children. Prior vaccination was evident in only 5 pediatric cases. The etiology of epiglottitis was similar across groups. Children had significantly higher chances of receiving epinephrine (34.78% vs. 8.82%), undergoing intubation (82.60% vs. 20.58%), being admitted to the ICU (56.52% vs. 17.64%), and having complications (47.82% vs. 14.70%), compared to adults”.  Additionally, we reworded some part to maintain appropriate word count (250 words) for the Abstract. |
| How could the differences in blood cultures influence the optimisation of diagnostic protocols | This is a great question!  It’s definitely one of the main components of the management plan of suspected epiglottitis patients (until a diagnosis of non-infectious epiglottitis can be reached).  We added a paragraph in the discussion section to highlight the role of blood cultures in this regard. | In the discussion section (page 10-11), we added this paragraph: “The disparity in blood culture performance between the two groups might reflect differing clinical approaches or variations in symptom severity. Understanding these differences is key to optimizing diagnostic protocols. Pediatric patients had a higher rate of blood culture performance (39.13% vs. 5.88% in adults), highlighting the need to rule out secondary bacterial infections more aggressively in this age group. Implementing routine blood cultures in pediatric patients with severe symptoms can facilitate early identification and appropriate treatment of concurrent infections, thereby improving patient outcomes. For adults, a more selective approach based on clinical presentation and risk factors may prevent unnecessary antibiotic use and associated resistance. These variations suggest the necessity for age-specific clinical guidelines that balance thorough evaluation in high-risk pediatric patients with a judicious approach in adults, ultimately enhancing patient care and resource utilization.” |
| How do we know that the increased intubation and epinephrine represents more aggressive disease rather than more aggressive management? | Thank you for this question!  The observed increase in intubation and epinephrine use in pediatric cases of non-infectious epiglottitis compared to adults could suggest either more aggressive disease or more aggressive management. The increased rate of both interventions (intubation: 82.60% vs. 20.58%, epinephrine: 34.78% vs. 8.82%) in the pediatric age group may be indicative of the inherently more severe presentation in children, who are more susceptible to rapid airway obstruction due to their anatomically smaller airways and different immune responses. However, it is also possible that clinicians adopt a more aggressive management approach in pediatric patients to preempt potential complications. The higher incidence of acute symptoms such as stridor and drooling in children may prompt earlier and more intensive interventions. | Under the discussion section, we added the following paragraph: “The observed increase in intubation and epinephrine use in pediatric cases of non-infectious epiglottitis compared to adults could suggest either more aggressive disease or more aggressive management. The increased rate of both interventions (intubation: 82.60% vs. 20.58%, epinephrine: 34.78% vs. 8.82%) in the pediatric age group may be indicative of the inherently more severe presentation in children, who are more susceptible to rapid airway obstruction due to their anatomically smaller airways and different immune responses. However, it is also possible that clinicians adopt a more aggressive management approach in pediatric patients to preempt potential complications. The higher incidence of acute symptoms such as stridor and drooling in children may prompt earlier and more intensive interventions.” |
| This is an interesting report, however the associations drawn need to conclude with a summary of clinical relevance. | Thank you for your suggestion. We added a new section highlighting the clinical relevance of our findings. | We added a new subsection in the Discussion section names “Clinical Relevance” as follows: “This study highlights significant age-related differences in the presentation and management of non-infectious epiglottitis. Pediatric patients demonstrate more severe clinical presentations, necessitating more frequent interventions such as intubation and epinephrine administration. These findings underscore the critical need for age-specific diagnostic and management protocols to improve patient outcomes. For pediatric patients, more aggressive monitoring and timely intervention are essential to prevent rapid airway obstruction and associated complications. In contrast, a more selective approach in adults may reduce unnecessary interventions and healthcare costs. Recognizing these differences and tailoring treatment strategies accordingly can lead to better resource utilization, more effective management, and ultimately improved patient care across different age groups.” |
