# Supplementary material for: Does patients’ age predict their clinical outcomes following non-infectious epiglottitis? A systematic review
Source: PLoS One. 2025 Feb 10;20(2):e0318648. doi: 10.1371/journal.pone.0318648 (PMC11809843; doi:10.1371/journal.pone.0318648)
Supplement: Attachment — Submitted filename: RESPONSE TABLE.docx [file pone.0318648.s008.docx]

**RESPONSE TABLE**

**Manuscript ID:** PONE-D-24-03809R1

**Manuscript Title:** Does Patients’ Age Predict Their Clinical Outcomes Following Non-Infectious Epiglottitis? A Systematic Review

| **Raised Comment** | **Authors’ Response** | **Applied Edits** |
| --- | --- | --- |
| The study was done through a systematic literature search which included studies from inception until December 22nd, 2023. The study aimed at analyzing differences in clinicodemographic characteristics, management strategies and clinical outcomes between pediatric and adult cases of non-infectious epiglottitis. The study involved the search of 4 databases, which yielded 40 studies and reported 57 individual patient data.  Overall, it is a well-thought out and executed research manuscript capable of contributing to the body of knowledge regarding the management of non-infectious epiglottitis. However, I have the following observations,  1. Methods: The study utilized mostly studies that were rated as being good quality. However, six of the studies utilized in the study were deemed fair quality. A factor that might have affected the overall quality of the manuscript. | We greatly appreciate your detailed feedback and insightful comments.  We agree that the inclusion of fair-quality studies could potentially affect the interpretation of our findings. However, this study employs an individual patient data meta-analysis, where sensitivity analyses, as conducted in traditional meta-analyses, are not feasible. Removing six cases from a dataset of 57 cases (3 adults and 3 children) would significantly reduce the statistical power of the analysis and might introduce further bias, as the excluded cases represent real-world variations in the quality of reporting. To address this concern, we included a clear statement about this limitation in the Discussion section. | Under the Discussion section, we added this sentence: “The inclusion of six fair-quality studies, while necessary to maximize the dataset in this rare condition, may introduce some variability. However, given the predominance of good-quality studies, we believe the findings remain robust and reflective of clinical realities.” |
| 2. References: The referencing style appears generally fine. However, it appears the conference proceedings cited (references 22) was not properly cited. The approach and checklist suggested by the University of Queensland library (guides.library.uq.edu.au), might be helpful. | Thank you for your suggestion. However, it appears that the provided link contains guidelines and tools for clinical practice rather that citation output style.  That being said, we revised the output style for this reference and updated it according to the journal’s style using EndNote. | Citation #22 is the same as before but the output style was corrected as per the journal’s style as follows:  22. Garg G, Sawhney H, Gandhi D, Bhatt N, Rosovsky M. Foreign body (toothpick) causing epiglottitis. Proceedings (Baylor University Medical Center). 2020;33(2):231-2. |
| 3. The phrasing of the language used in the entire document could benefit from a more professional grammatical input. | Thank you for your feedback. We revised the manuscript and improved some aspects of it. | The following grammatical improvements were carried out:  **Abstract**  **Current**: "The search of four databases yielded 57 individual patient data, all of whom had non-infectious epiglottitis."  **Updated**: "A systematic search of four databases identified 57 individual patient records, all diagnosed with non-infectious epiglottitis."  **Current**: "Differences regarding clinicodemographic data, management plan, and clinical outcomes were analyzed."  **Updated**: "Differences in clinicodemographic characteristics, management strategies, and clinical outcomes were analyzed."  **Introduction**  **Current**: "The management of this condition is complex, necessitating a careful balance between therapeutic intervention and watchful waiting..."  **Updated**: "Managing this condition is complex and requires balancing therapeutic intervention with watchful observation..."  **Current**: "Other factors, including the specific cause of epiglottitis and presenting symptoms, also play a crucial role in determining the need for airway intervention."  **Updated**: "Factors such as the specific cause of epiglottitis and presenting symptoms significantly influence the need for airway intervention."  **Materials and Methods**  **Current**: "The detailed search criteria used in identifying relevant cases are provided in S1 Table."  **Updated**: "Detailed search criteria for identifying relevant cases are presented in S1 Table."  **Current**: "The literature search was carried out by our institution’s librarian."  **Updated**: "Our institution’s librarian conducted the literature search."  **Results**  **Current**: "Compared to adult cases, children with non-infectious epiglottitis were significantly more likely to present with stridor..."  **Updated**: "Children with non-infectious epiglottitis were significantly more likely than adults to present with stridor..."  **Discussion**  **Current**: "The observed increase in intubation and epinephrine use in pediatric cases of non-infectious epiglottitis compared to adults could suggest either more aggressive disease or more aggressive management."  **Updated**: "The higher rates of intubation and epinephrine use in pediatric cases compared to adults may indicate either a more aggressive disease course or a more proactive management approach."  **Current**: "The high usage of broad-spectrum therapies points to a potential area for guideline refinement, emphasizing etiology-specific treatments."  **Updated**: "The frequent use of broad-spectrum therapies highlights a potential need for refining guidelines to emphasize etiology-specific treatments." |
| 4. I found the title, study design, statistics, figures and tables quite satisfactory. | Thank you. Your feedback has been greatly appreciated. | No edits were applied. |
